# Two Pear Glutathione *S*-Transferases Genes Are Regulated during Fruit Development and Involved in Response to Salicylic Acid, Auxin, and Glucose Signaling

**DOI:** 10.1371/journal.pone.0089926

**Published:** 2014-02-25

**Authors:** Hai-Yan Shi, Zheng-Hong Li, Yu-Xing Zhang, Liang Chen, Di-Ying Xiang, Yu-Feng Zhang

**Affiliations:** 1 College of Horticulture, Agricultural University of Hebei, Baoding, China; 2 Key Laboratory of Plant Germplasm Enhancement and Specialty Agriculture and Wuhan Botanical Garden, Chinese Academy of Sciences, Wuhan, China; National Taiwan University, Taiwan

## Abstract

Two genes encoding putative glutathione *S*-transferase proteins were isolated from pear (*Pyrus pyrifolia*) and designated *PpGST1* and *PpGST2*. The deduced PpGST1 and PpGST2 proteins contain conserved Glutathione S-transferase N-terminal domain (GST_N) and Glutathione S-transferase, C-terminal domain (GST_C). Using PCR amplification technique, the genomic clones corresponding to *PpGST1* and *PpGST2* were isolated and shown to contain two introns and a singal intron respectively with typical GT/AG boundaries defining the splice junctions. Phylogenetic analysis clearly demonstrated that PpGST1 belonged to Phi class of GST superfamilies and had high homology with apple MdGST, while PpGST2 was classified into the Tau class of GST superfamilies. The expression of *PpGST1* and *PpGST2* genes was developmentally regulated in fruit. Further study demonstrated that *PpGST1* and *PpGST2* expression was remarkably induced by glucose, salicylic acid (SA) and indole-3-aceticacid (IAA) treatments in pear fruit, and in diseased fruit. These data suggested that *PpGST1* and *PpGST2* might be involved in response to sugar, SA, and IAA signaling during fruit development of pear.

## Introduction

Glutathione *S*-transferases (GSTs; EC 2.5.1.18) are a superfamily of multifunctional enzymes that catalyze the nucleophilic conjugation of reduced tripeptide glutathione (GSH; g-Glu-Cys-Gly) into a variety of hydrophobic and electrophilic compounds to direct them to specific sites both intra- and extracellularly. GSTs protect tissues against oxidative stress or from toxic products produced during xenobiotic metabolism [1]–[3]. Additionally, plant GSTs are involved in development [2], [4].

Plant GSTs have been mainly divided into eight classes: phi, tau, lambda, theta, zeta, EF1Bg, dehydroascorbate reductase (DHAR), and tetrachlorohydroquinone dehalogenase (TCHQD) [5]–[8]. Among these, phi, tau, DHAR, and lambda GSTs are specific to plants. Recently, two new GST classes, hemerythrin and iota, were identified in *Physcomitrella patens* that is a nonvascular representative of early land plants [9]. Phi and tau GSTs are the most abundant in plant and are involved mainly in xenobiotic metabolism [3], [10]. However, evidence to substantiate plant development has been limited.

Plant *GST* genes form a large gene family. *GSTs* have been identified in some plants, such as tomato [11], Arabidopsis (*Arabidopsis thaliana*) [12], poplar [6], rice (*Oryza sativa*) [8], and barley [13]. The barley *SIGST* gene might play an important role during leaf senescence [13]. Moreover, plant *GST*s can be induced by a wide variety of phytohormones, including salicylic acid (SA), auxin, ethylene, methyl jasmonate, and abscisic acid (ABA) [14]–[17]. That all these hormones regulate many aspects of plant development implies that plant *GSTs* may play crucial roles in plant development as well. The study aims to elucidate the regulation of the pear *GST* genes during fruit ripening and senescence, under glucose, SA and auxin treatment, and disease resistance, which would provide valuable information for fruit senescence, disease resistance and sugar signaling studies in pear.

## Materials and Methods

### Collection of Plant Materials

Pear (*Pyrus pyrifolia* Nakai. cv. Whangkeumbae) fruit were harvested at 30, 60, 90, 120, 130, 140, 150 d after full bloom from the experimental farm of horticulture plants of Agricultural University of Hebei, China. The fruit, harvested at 150 d after full bloom that is natural harvest date, were placed for 10, 20, and 30 days at room temperature for the collection of 10, 20, and 30 d after harvest fruit respectively. The diseased fruit and the controls were chosen from the above 10 d after harvest pear fruit. The mesocarp of the pears was collected for further study. The other tissues (such as shoots, young leaves, petals, and anthers) were derived from the same pear trees of the local orchard. These samples were frozen immediately in liquid nitrogen, and then stored at –80°C for RNA isolation.

### Fruit Treatment

The mesocarp discs of pear fruit collected at 20 d after harvest were prepared with cork borer. The diameter of these discs was 6 mm and the thickness was 2 mm. The mesocarp discs of pear were dipped in distilled water solutions containing 0.002, 0.02, 0.2 and 2 mM SA for 12 h for treatment, respectively. Untreated control mesocarp discs were dipped into distilled water for 12 h. The part of the mesocarp discs were also treated with 0.2 mM SA for 3, 6, 12, and 24 h respectively. Untreated mesocarp discs were dipped into distilled water immediately as control.

The mesocarp discs of pear fruit collected at 10 d after harvest were prepared with cork borer. The diameter of these discs was 6 mm and the thickness was 2 mm. For glucose treatments, a part of the mesocarp discs were subjected to 5%, 10%, 15%, and 20% of glucose solutions for 12 h, respectively. Some of the mesocarp discs were subjected to 10% of glucose solution. Samples were then collected in 0, 3, 6, 12, and 24 h intervals, respectively. The other part of the mesocarp discs were treated with 0.2 mM IAA for 3, 6, 12, 24, and 36 h respectively. Untreated mesocarp discs were dipped into distilled water immediately as control.

Twenty mesocarp discs from 30 fruit of the same pear trees were prepared with cork borer and cultured for SA, glucose, and IAA treatments. Total RNA was isolated from the treated mesocarp discs and controls of pear. All the mesocarp disc preparation, treatments, and RNA isolation were repeated three times. The data were input into SPSS software, and t test of independent samples was performed for statistical inference.

### Construction of Pear Fruit cDNA Library and Isolation of *PpGST1* and *PpGST2* cDNAs

Total RNA was extracted from pear fruit collected at 90, 120, 130, 140 and 150 d after full bloom, and 10, 20 and 30 d after harvest. Poly(A)^+^ mRNA was prepared from a pool of fruit total RNA by using an mRNA purification kit (Qiagen). Complementary DNA was synthesized and cloned into the *Eco*R I–*Xho* I sites of the ZAP express vector and packaged using a ZAPcDNA Gigapack Gold III cloning kit (Stratagene) according to the manufacturer’s instruction.

More than 3,000 cDNA clones were randomly selected from the pear fruit cDNA library for sequencing. Two *PpGST* clones with complete sequences were identified. The corresponding *PpGST* genes were amplified from the genomic DNA of pear by PCR, using *Pfu* DNA polymerase and gene-specific primers that were designed according to the sequence of each *PpGST* cDNA. In total, two *PpGST* genes were obtained.

### RNA Isolation and Quantitative RT-PCR Analysis

Total RNA was isolated from shoots, leaves, petals, anthers, and developing mesocarp of pear by the method described previously [18]. A 2–4 g aliquot of each pear tissue was randomly collected from 3–10 plants for RNA isolation. The concentration and purity of total RNA were identified by NanoDrop spectrophotometry and agarose gel electrophoresis. RNA samples were stored at –80°C until use.

Expression profiling of the *PpGST* genes in different pear tissues (such as shoots, leaves, petals, and anthers) and during different stages of fruit development was carried out by quantitative RT-PCR using the fluorescent intercalating dye SYBR-Green in the detection system (Mastercycler ep realplex 4, Eppendorf AG, Hamburg, Germany). A pear β-actin gene was used as a standard control in the RT-PCRs. A two-step RT-PCR procedure was performed in all experiments using a previously described method [18]. In brief, total RNA was reverse transcribed into cDNA and used as a template in PCRs with gene-specific primers (*PpGST1* P1: 5′-GAGGAAAGATTGTCAAAAAGCAAA-3′; P2: 5′-ACGTGCGGAACTATATCATATTGA-3′. *PpGST2* P1: 5′-CTTATCTTTTGGATGGATAGCATTC-3′; P2: 5′-CGTGTTTGGTTCATGTATATATATTA-3′). Quantitative RT-PCR was performed using SYBR-Green Real-time PCR Master Mix according to the manufacturer’s instructions (Toyobo Co. Ltd, Osaka, Japan). The Ct (cycle threshold), defined as the PCR cycle at which a statistically significant increase of reporter fluorescence was first detected, was used as a measure of the starting copy number of the target gene. The relative quantity of the target *PpGST* expression level was determined using the comparative Ct method [18]. To achieve optimal amplification, PCR conditions for every primer combination were optimized for annealing temperature, and PCR products were verified by melting curve analysis and confirmed on an agarose gel. Mean values and standard deviation (bar) were calculated in three independent experiments with three biological replicates of tissue materials, and the data were normalized with the relative efficiency of each primer pair.

### DNA Sequencing and Protein Analysis

The sequences of the isolated pear *GST* genes (cDNAs) and their deduced proteins were analyzed using DNAstar software (DNAstar Inc., Madison, WI, USA). The conserved domain was determined by NCBI Conserved Domain Search (http://blast.ncbi.nlm.nih.gov/Blast.cgi). Protein sequence homology analysis was performed with ClustalW (http://www.ebi.ac.uk/clustalw/) and protein motif analysis was performed through using motif scan (http://myhits.isb-sib.ch/cgi-bin/motif_scan). Seventeen GST protein sequences from different plants were aligned with the ClustalX program, and then the evolutionary relationships of the seventeen GST proteins were determined by MEGA3.1 software, which were based on minimum evolution-rule consensus from 1000 bootstrap replicates.

## Results

### Isolation and Characterization of *PpGST1* and *PpGST2*

**Figure 1:**
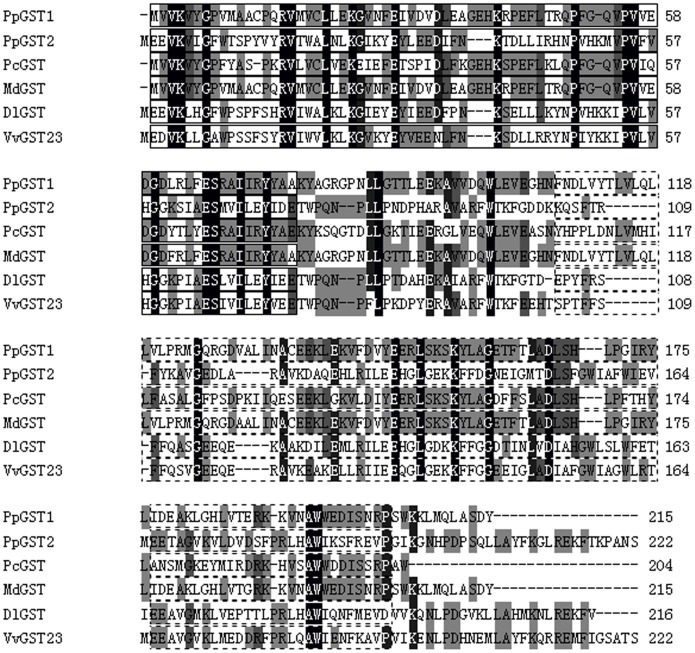
Sequence alignment among the six plant GST proteins. Sequences of plant GSTs were aligned. Identical amino acid sequences are highlighted in black and sequence similarities in grey. Glutathione S-transferase N-terminal (GST_N) domains are shown in the boxes. Dotted lines represent glutathione S-transferase C-terminal (GST_C) domains. The accession numbers of plant GST proteins in GenBank are: *Pyrus pyrifolia* PpGST1 (KF730655), PpGST2 (KF730656), *Pyrus communis* PcGST (ABI79308), *Malus domestica* MdGST (AEN84869), *Dimocarpus longan* DlGST (AFF18813), and *Vitis vinifera* VvGST23 (XP_002267691).

By randomly selecting and sequencing pear fruit cDNA library, two cDNAs encoding GST proteins were identified. The isolated cDNAs were designated as *PpGST1* and *PpGST2*, accession numbers in GenBank: KF730655 and KF730656. *PpGST1* cDNA encodes a GST homolog comprising 215 amino acids and shares relatively high homology (98% identity) with apple *MdGST* (*Malus domestica*, AEN84869) at amino acid level. *PpGST2* cDNA encodes a protein with 222 amino acids and shares relatively high homology (60% identity) with grape *VvGST23* (*Vitis vinifera*, XP_002267691) and longan *DlGST* (*Dimocarpus longan*, AFF18813) at amino acid level. However, the deduced PpGST1 and PpGST2 proteins show 17% amino acid sequence identity to each other. PpGST proteins contain conserved Glutathione S-transferase N-terminal domain (GST_N) and Glutathione S-transferase, C-terminal domain (GST_C), just as some other GST proteins (Figure 1).

**Figure 2:**
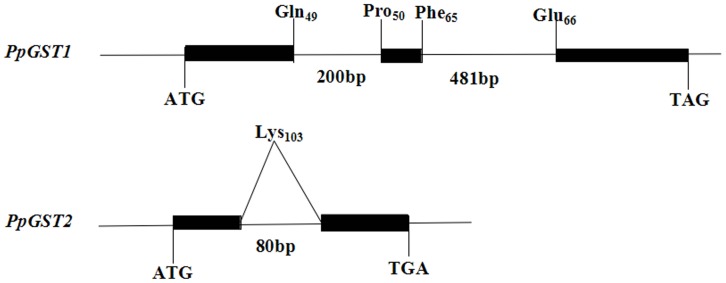
The structures of the two *PpGST* genes. Exons are denoted by black boxes. Introns, 5′-flanking regions, and 3′-UTRs are denoted by lines. The length of the intron in base pairs is indicated. The position of substitution is denoted by a diagonal line.

Subsequently, the genomic DNA sequences of *PpGST* genes were isolated in pear. Compared with its cDNA sequence, we found that *PpGST1* gene contains two introns in its open reading frame (ORF). The first intron is positions between codon 49 (Gln49) and codon 50 (Pro50) and is 200 bp in length, while the second intron inserts between codon 65 (Phe65) and codon 66 (Glu66) and is 481 bp in length in *PpGST1* gene. However, *PpGST2* gene contains a single intron in its ORF. The intron of *PpGST2* gene is located within codon 103 (Lys103) and is 80 bp in length (Figure 2).

### Phylogenetic Relationships of PpGSTs with the other Plant GST Proteins

**Figure 3:**
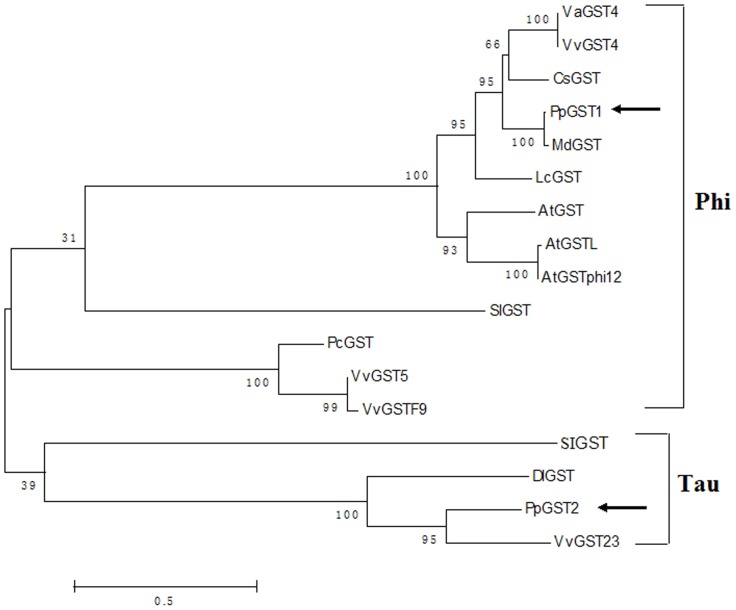
Phylogenetic relationships of PpGST proteins to other GST proteins. The minimum evolution tree was constructed in MEGA3.1 from 1000 bootstrap replicates. The accession numbers of plant GST proteins in GenBank are: PpGST1 (*Pyrus pyrifolia*, KF730655), PpGST2 (KF730656), VaGST4 (*Vitis amurensis*, ACN38271), VvGST4 (*Vitis vinifera*, XP_002271709), CsGST (*Citrus sinensis*, ABA42223), MdGST (*Malus domestica*, AEN84869), LcGST (*Litchi chinensis*, ABR15777), AtGST (*Arabidopsis thaliana*, NP_186969), AtGSTL (BAD89984), AtGSTphi12 (NP_197224), SlGST (*Solanum lycopersicum*, NP_001234088), PcGST (*Pyrus communis*, ABI79308), VvGST5 (ABW34390), VvGSTF9 (XP_002283209), SIGST (*Hordeum vulgare*, AB207242), DlGST (*Dimocarpus longan*, AFF18813), and VvGST23 (XP_002267691).

Analysis of the phylogenetic relationships between PpGSTs and other GST proteins previously reported in plants is shown in Figure 3. The PpGST1 protein, together with VaGST4, VvGST4, CsGST [19], MdGST, LcGST, AtGST, AtGSTL, AtGSTphi12, SlGST [20], PcGST [21], VvGST5, and VvGSTF9 form the first subgroup of the plant GST tree and belong to Phi class of GST superfamilies. Moreover, PpGST1 has the highest homology with apple MdGST. However, PpGST2, SIGST [13], DlGST, and VvGST23 occupy another clade of the tree and are classified into the Tau class of GST superfamilies, which suggests that PpGST1 might have diverged earlier from these GSTs during evolution.

### PpGST Gene Expression is Regulated during Fruit Development

**Figure 4:**
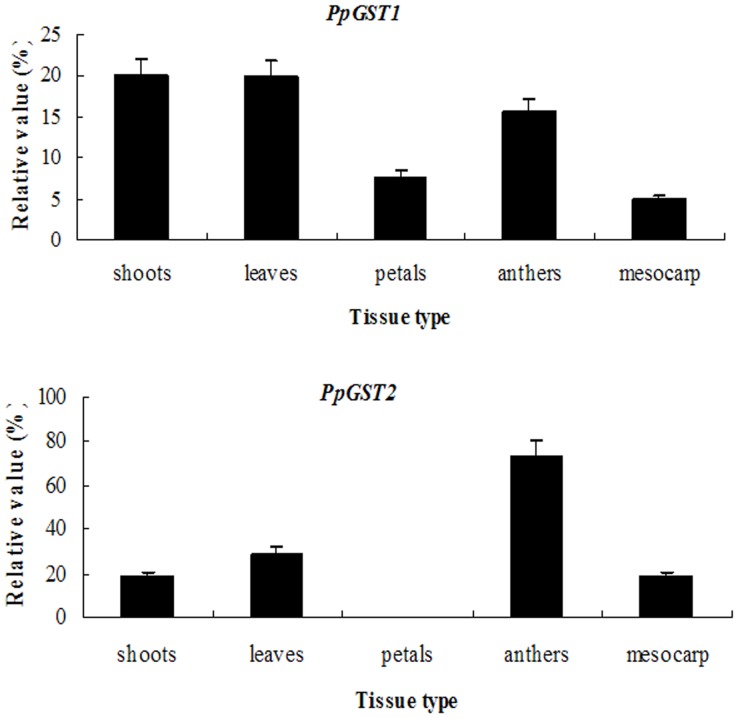
Quantitative RT-PCR analysis of expression of *PpGST1* and *PpGST2* genes in pear. Expression of *PpGST1* and *PpGST2* genes in different pear tissues. Total RNAs were isolated from different tissues [(i) shoots; (ii) young leaves; (iii) petals; (iv) anthers; and (v) mesocarp]. Relative value of expression of *PpGSTs* in pear tissues is shown as a percentage of β-actin expression activity. Mean values and SD (bar) were shown from three independent experiments. Values shown are means±SD for three replicates.

To explore the *PpGST* gene expression pattern, quantitative RT-PCR analysis was performed. *PpGST1* is preferentially expressed in pear shoots and young leaves, and moderate expression was found in anthers, but relatively weak signals were detected in petals and mesocarp of fruit (Figure 4). As shown in Figure 4, Transcripts of *PpGST2* were preferentially accumulated at relatively high levels in anthers, and at moderate to low levels in leaves, shoots, and mesocarp of fruit. However, no expression signal was detected in petals.

**Figure 5:**
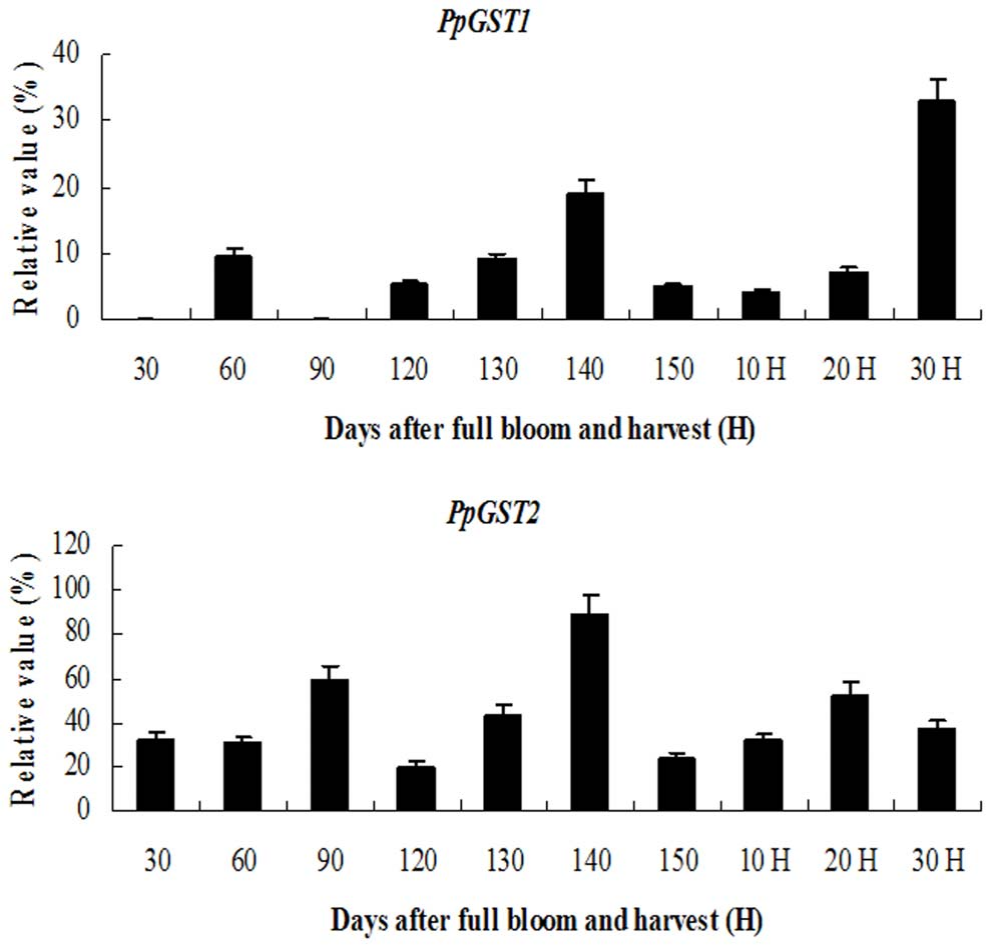
Quantitative RT-PCR analysis of expression of *PpGST1* and *PpGST2* genes during fruit development. Relative value of expression of *PpGSTs* in pear tissues is shown as a percentage of β-actin expression activity. Mean values and SD (bar) were shown from three independent experiments. Values shown are means ±SD for three replicates.

To investigate whether expression of *PpGSTs* is regulated during fruit development, further analysis for the expression pattern of *PpGSTs* during fruit development was performed. The experimental results (Figure 5) revealed that *PpGST1* transcripts were detected at relatively low level in 30–130 d after full bloom fruit. The expression activity of *PpGST1* increased to a relative high level in 140 d after full bloom fruit. With the fruit ripening, the expression of *PpGST1* gene decreased to a relative low level in 150 d after full bloom fruit. With the fruit softening and senescence, the expression of *PpGST1* gene remained to a relative low level in 10–20 d after harvest fruit. However, the expression activity of *PpGST1* increased to the highest level in 30 d after harvest fruit. These results suggested that the *PpGST1* gene might play crucial roles during fruit ripening and senescence of pear. The expression activity of *PpGST2* gene remained higher levels than that of *PpGST1* during the whole fruit development (Figure 5). Moreover, *PpGST2* transcripts were detected at relatively high level in 140 d after full bloom fruit as shown in Figure 5, which suggesting that *PpGST2* gene might be involved in pear fruit ripening. Taken the results together, the two *PpGST* genes might play important roles during fruit ripening and senescence of pear.

### Expression of *PpGST* Genes in Fruit is Regulated by SA, IAA, and Disease

**Figure 6:**
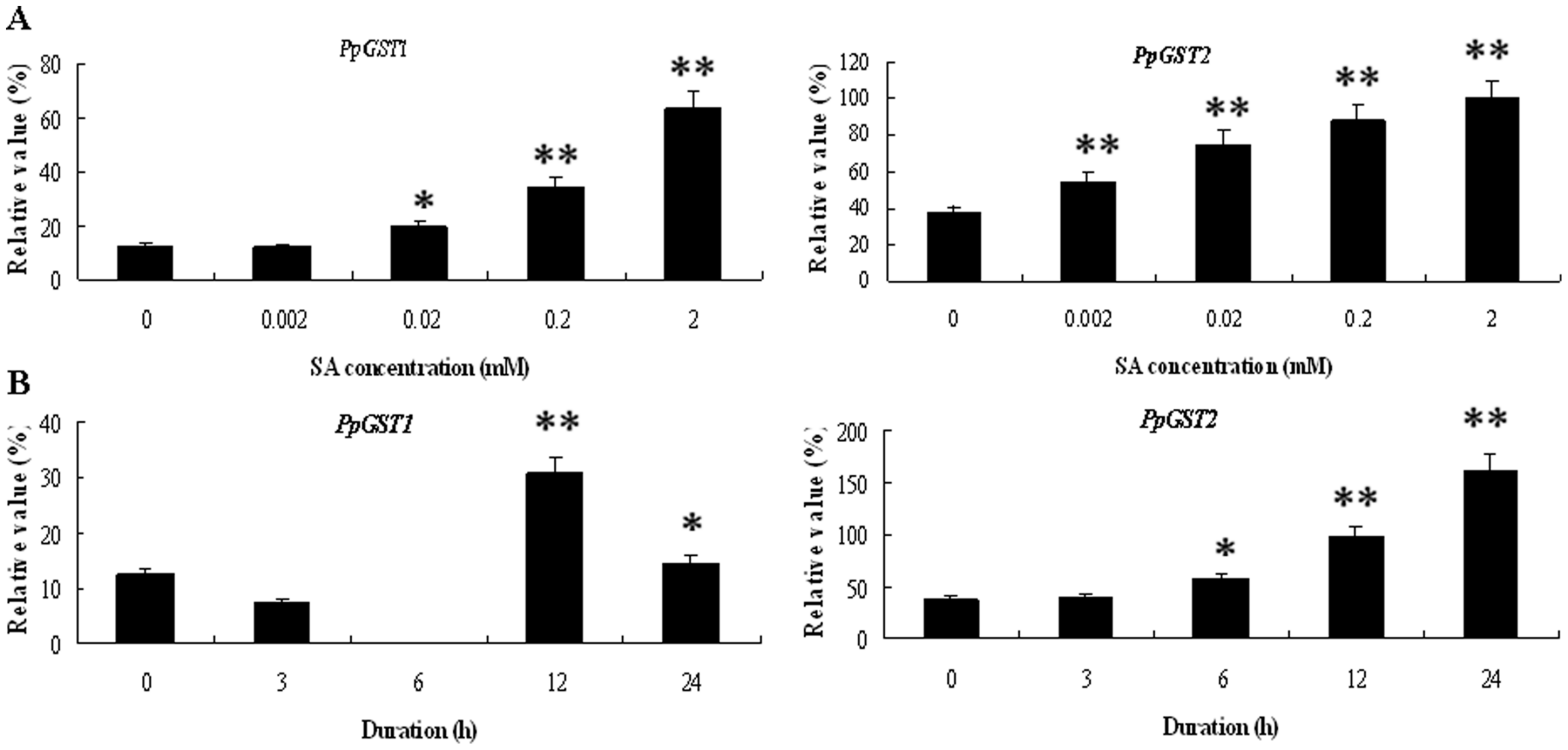
Quantitative RT-PCR analysis of expression of *PpGST* genes in fruit under SA treatments. (**A**) Quantitative RT-PCR analysis of expression of *PpGST* genes in fruit under SA treatment for 12 h. Relative values of expression of *PpGST* genes in 20 d after harvest fruit treated with 0, 0.002, 0.02, 0.2 and 2 mM SA for 12 h, respectively, are shown as a percentage of β-actin expression activity. (**B**) Quantitative RT-PCR analysis of expression of *PpGST* genes in fruit under 0.2 mM SA treatment. Relative values of expression of *PpGST* genes in 20 d after harvest fruit treated with 0.2 mM SA for 0, 3, 6, 12, and 24 h, respectively, are shown as a percentage of β-actin expression activity. Mean values and standard errors (bar) were shown from three independent experiments. Values shown are means ±SD for three replicates. Independent t tests for equality of means demonstrated that there was significant difference (*P value<0.05) or very significant difference (**P value<0.01) between control and treated fruit.

To investigate whether the expression of the isolated *PpGST* genes was regulated by SA, mesocarp of 20 d after harvest fruit was subjected to SA treatments. Total RNAs were isolated from the treated and un-treated mesocarp discs of pear, and then reversely transcribed into cDNAs for quantitative PCR analysis. As shown in Figure 6A, *PpGST1* gene was induced significantly by 0.02, 0.2 and 2 mM SA for 12 h, but was not induced significantly by 0.002 mM SA. *PpGST2* gene was induced significantly by every concentration of SA for 12 h. These results suggested that the induction might relate to the high concentration of SA. However, *PpGST1* gene was induced by 0.2 mM SA for 12 and 24 h, while *PpGST2* was induced by 0.2 mM SA for 6, 12, and 24 h (Figure 6B); revealing the two pear *GST* genes have different expression patterns in response to SA.

**Figure 7:**
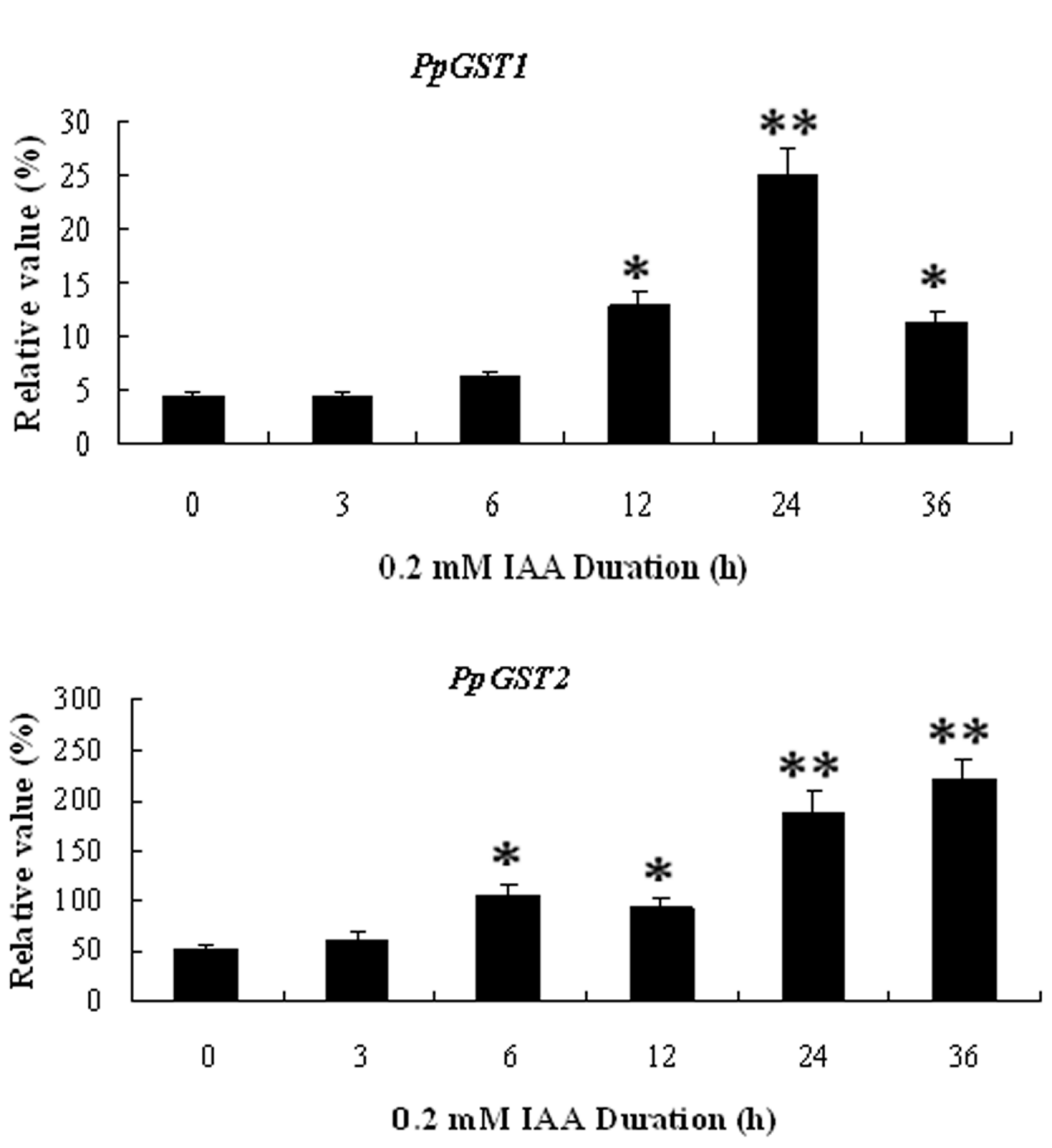
Quantitative RT-PCR analysis of expression of *PpGST* genes in fruit under 0.2 mM IAA treatments. Expression of *PpGST1* and *PpGST2* was analysed in 10 d after harvest fruit at 0.2 mM IAA treatment for 3, 6, 12, 24, and 36 h. Relative value of *PpGST1* and *PpGST2* expression in pear fruit including different time courses of 0.2 mM IAA treatment is shown as a percentage of β-actin expression activity. Mean values and standard errors (bar) were shown from three independent experiments. Values shown are means±SD for three replicates. Independent t tests for equality of means demonstrated that there was significant difference (*P value<0.05) or very significant difference (**P value<0.01) between control and treated fruit.

**Figure 8:**
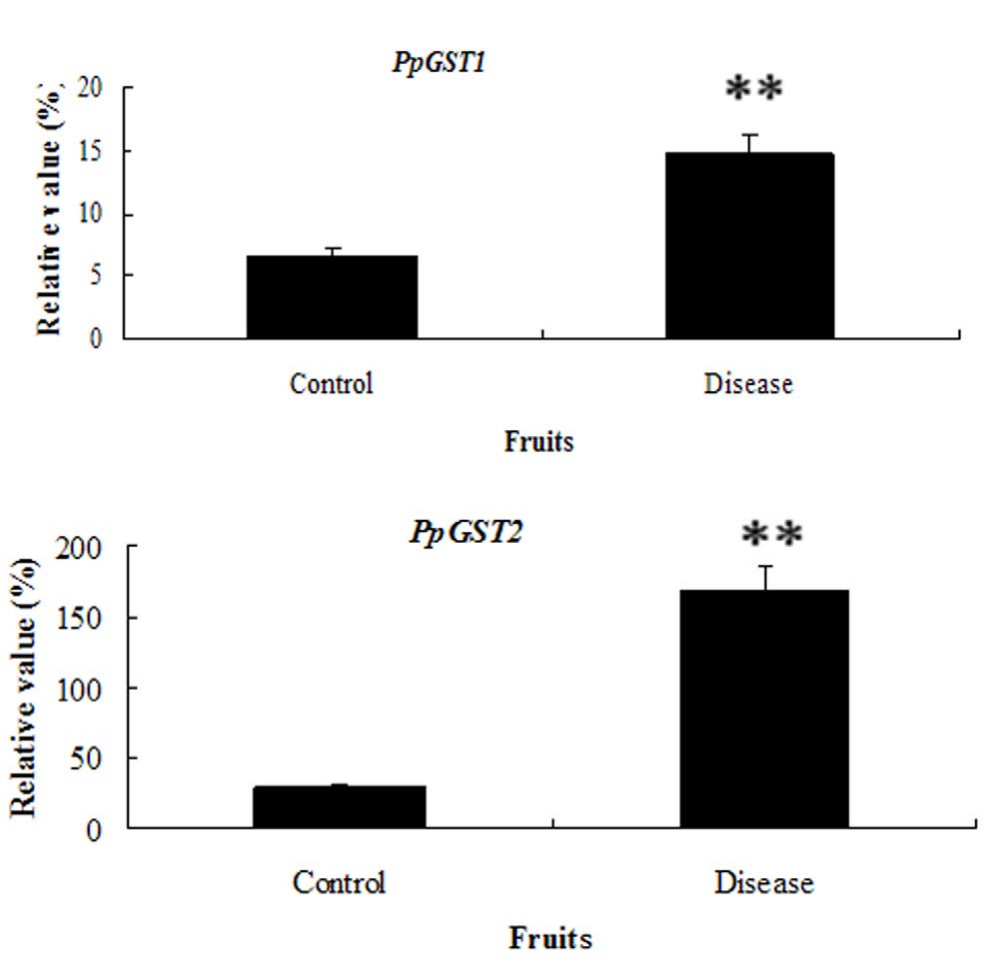
Quantitative RT-PCR analysis of expression of *PpGST* genes in diseased fruit. Relative values of *PpGST* gene expression in 10 d after harvest diseased fruit of pear are shown as a percentage of β-actin expression activity. Mean values and standard errors (bar) were shown from three independent experiments. Values shown are means ±SD for three replicates. Independent t tests for equality of means demonstrated that there was very significant difference (**P value<0.01) between control and treated fruit.

To investigate whether the expression of the isolated *PpGST* genes was regulated by IAA, mesocarp of 10 d after harvest fruit was subjected to IAA treatments. *PpGST1* gene was induced by 0.2 mM IAA for 12, 24 and 36 h, while *PpGST2* was induced by 0.2 mM IAA for 6, 12, 24, and 36 h (Figure 7). Additionally, the expression of *PpGSTs* was up-regulated in 10 d after harvest diseased fruit (Figure 8). These results suggested that *PpGST* genes might be involved in response to IAA and disease signaling during fruit development of pear.

### Expression of *PpGST* Genes in Fruit is Regulated by Glucose

**Figure 9:**
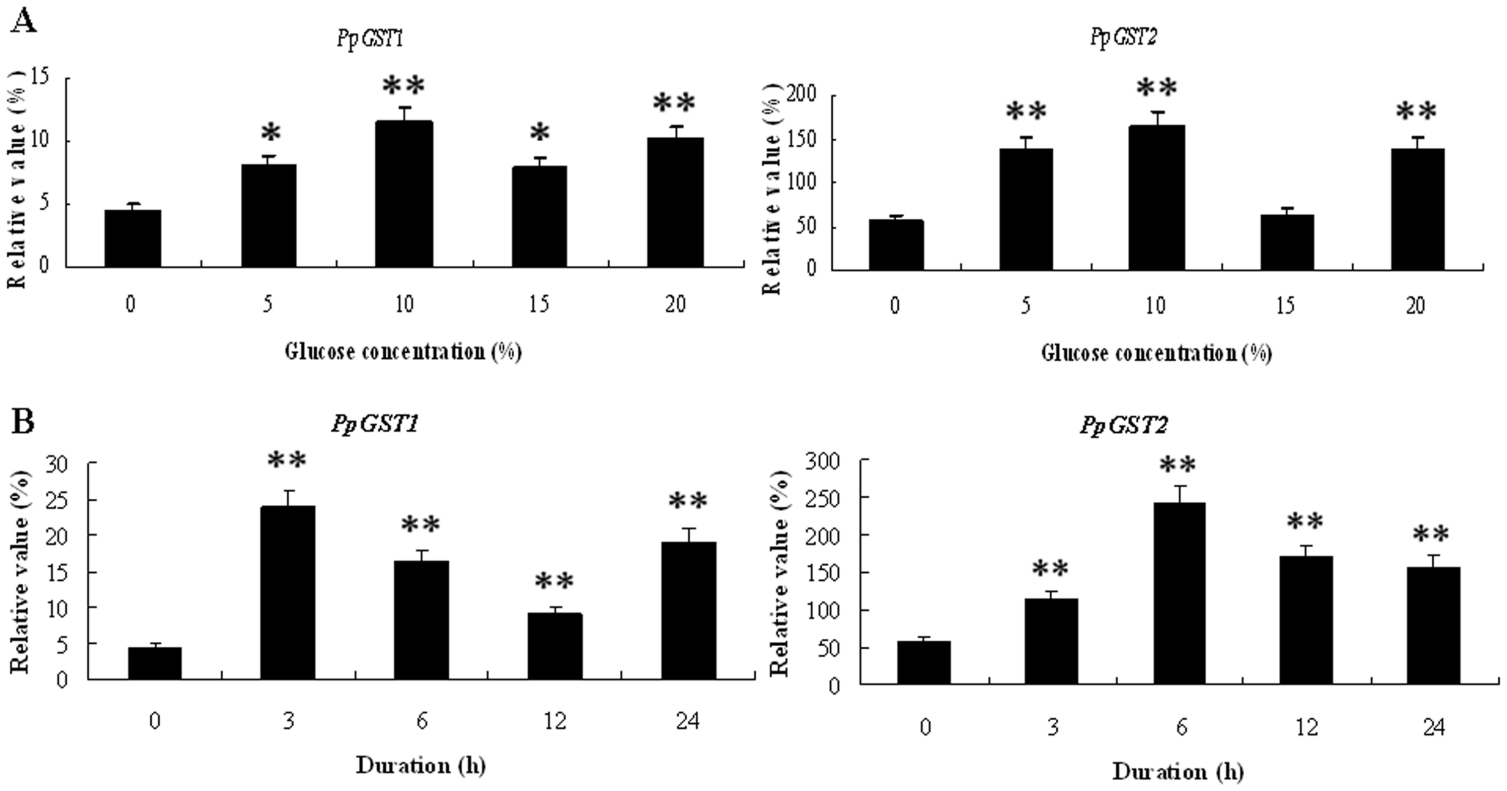
Quantitative RT-PCR analysis of expression of *PpGST* genes in fruit under glucose treatments. (**A**) Quantitative RT-PCR analysis of expression of *PpGST* genes in fruit under glucose treatment for 12 h. Relative values of expression of *PpGST* genes in 10 d after harvest fruit treated with 0, 5%, 10%, 15%, and 20% glucose for 12 h, respectively, are shown as a percentage of β-actin expression activity. (**B**) Quantitative RT-PCR analysis of expression of *PpGST* genes in fruit under 10% glucose treatments. Relative values of expression of *PpGST* genes in 10 d after harvest fruit treated with 10% glucose for 0, 3, 6, 12, and 24 h, respectively, are shown as a percentage of β-actin expression activity. Mean values and standard errors (bar) were shown from three independent experiments. Values shown are means ±SD for three replicates. Independent t tests for equality of means demonstrated that there was significant difference (*P value<0.05) or very significant difference (**P value<0.01) between control and treated fruit.

To study the effects of glucose on the activities of *PpGST* genes in pear fruit, we examined the expression profiles of the *PpGST* genes in 10 d after harvest fruit treated with glucose. The experimental results showed that the transcript levels of *PpGST1* in 10 d after harvest fruit were significantly up-regulated by 5%, 10%, 15%, and 20% glucose for 12 h, while the transcript levels of *PpGST2* in 10 d after harvest fruit were significantly up-regulated by 5%, 10%, and 20% glucose for 12 h. The expression of *PpGST* genes was up-regulated to the highest level with 10% glucose (Figure 9A). With the increase of 10% glucose treatment time, the expression of *PpGST* genes was very significantly regulated in fruit (Figure 9B). The results suggested that *PpGST* genes might be involved in response to glucose signaling during fruit development of pear.

## Discussion

GSTs are soluble or loosely membrane-associated dimers with a monomeric size of 15–28 kDa, and together comprise 1–3.5% of total cellular protein [22]–[23]. N- and C-terminal functional domains of GSTs (GST_N and GST_C) seem to have evolved under a strong purifying selection (Ka/Ks <1) under different selective pressures [24]. In this study, PpGST1 (24.56 kD, pI 5.53) and PpGST2 (25.70 kD, pI 6.55) also share the GST_N and GST_C just like other GSTs, suggesting the two domains might play important roles for the function of the PpGSTs. Additionally, the *PpGST1* gene contained two introns and could be classified into Type I GSTs based on intron/exon structure alone. Type I GSTs, with two introns, have been identified in numerous plant species. For example, the grape *VvGST2* and *VvGST3* genes have contained two introns [25] just like the *PpGST1* gene in the study. Type II GSTs have been identified in wheat and carnation, with the gene in carnation containing nine introns [26]. The *PpGST2* contained a single intron and belong to Type III GSTs which containing a single intron and making up the second largest family of plant GSTs, including many of the GSTs originally identified as auxin-regulated proteins [27]–[28]. Both *VvGST1* and *VVGST5* contained a single intron [25] and were classified into Type III GSTs just like the *PpGST2* gene in the study.

In plants, *GST* expression is induced by phytohormones. Individual GSTs from Arabidopsis and other plants have been shown to be induced by SA [17], [29], auxin [16], [30], ethylene [16], [31]–[32], cytokinin, ABA [29], methyl jasmonate [15], and brassinosteroid [33]. The *SIGST* gene is classified into the Tau class of *GST* superfamilies, which is involved in stress responses [13]. Weak expression of *SIGST* was induced by indole-3-aceticacid (IAA) and 2, 4-D [13]. The expression of *PpGST* genes was regulated by plant hormones SA (Figure 6) and IAA (Figure 7), suggesting that *PpGST* genes might be involved in response to SA and IAA signaling during fruit senescense.

*GST* expression in plants is up-regulated in many different stress situations [14], [29]. A *GST* from tomato was shown to inhibit cell death and enhance oxidative stress-tolerance in yeast [11]. The *PpGST1* transcripts were mainly accumulated in 30 d after harvest fruit during the senescence of pear (Figure 5), which suggesting that *PpGST1* might play an important role in senescence of fruit. The expression of *GSTs* can also be induced by pathogen attack [34]. *GSTF8*, a particular Arabidopsis Phi class *GST*, is used as a marker for early stress/defense responses. *GSTF8* expression can be induced by microbial infection [35]–[36]. The expression of *PpGSTs* was induced in diseased fruit cell (Figure 8). Moreover, the expression relative vale of *PpGST2* in the diseased fruit was 3-fold more than that in the control, which suggesting that the gene might be involved in disease resistance during fruit ripening and senescence.

Expression profiling also indicated that the gene family plays a role not only in stress-related biological processes but also in the sugar-signaling pathway. The expression of five Tau *GST* genes was up-regulated under sucrose treatment based on microarray analysis, which suggesting that the Tau subfamily members in plants might play a role in sugar signaling [24]. *PpGST2*, a member of the Tau *GST* subfamily, was induced by glucose. Interestingly, *PpGST1*, a member of the Phi *GST* subfamily, was also induced by glucose, which suggesting that the Phi subfamily members in plants also might play a role in sugar signaling and these two pear *GST* genes might be involved in the sugar signaling pathway during fruit ripening and senescence. In the present study, we found that *PpGSTs* are induced by a number of intracellular and environmental factors including senescence, hormones, disease, and glucose. These results strongly suggested that *PpGSTs* were deeply involved in the metabolism during pear fruit senescence.
